# Effect of preheating on the degree of conversion, rheological behavior, and microhardness of resin composites: An in vitro ATR-FTIR and mechanical analysis

**DOI:** 10.4317/jced.64273

**Published:** 2026-08-28

**Authors:** Chinmay Dattatraya Bhat, Gowrish S Bhat, Vandana Sadananda

**Affiliations:** 1 Conservative Dentistry and Endodontics, AB Shetty Memorial Institute of Dental Sciences, Deralakatte, Mangaluru, 575018, Karnataka, India

**Keywords:** ATR-FTIR, degree of conversion, preheating, resin composites, rheological behavior, Vickers microhardness.

## Abstract

**Background:**

Preheating of resin composites is recommended as a strategy to enhance flowability, curing efficiency, and mechanical characteristics during restorative procedures. However, the influence of preheating on different resin matrix systems remains variable and material dependent. This study aimed to assess the impact of preheating on the degree of conversion, rheological behavior, and Vickers microhardness of resin composites with different resin matrix systems.

**Materials and Methods:**

Three light-cured resin composites comprising Bis-GMA-rich, UDMA-rich, and TEGDMA-rich formulations were evaluated under room temperature and preheated conditions. A total of 150 specimens were prepared and analyzed for degree of conversion using ATR-FTIR spectroscopy, rheological behavior using a rotational rheometer, and Vickers microhardness employing a microhardness indenter. Statistical comparisons were conducted with Student's t-test, one-way ANOVA, and Tukey HSD post hoc tests.

**Results:**

Preheating produced a statistically significant rise in the degree of conversion and Vickers microhardness values in all tested resin composite groups (p<0.05). TEGDMA-rich composites demonstrated the highest degree of conversion values, whereas UDMA-rich composites demonstrated the highest microhardness values. Rheological analysis demonstrated alterations in viscosity and flow behavior following preheating among the evaluated resin composites.

**Conclusions:**

Preheating improved the polymerization efficiency, flow characteristics, and microhardness of resin composites. Preheating may therefore be considered a beneficial clinical approach for improving the handling and mechanical performance of restorative composites.

## Introduction

Resin-based composites rank among the most commonly employed restorative materials in modern dentistry because of their favourable aesthetics, adhesive properties, and improved mechanical performance [1,2]. The sustained clinical durability of composite restorations is closely related to adequate polymerisation and optimal physical properties following curing.

The resin matrix composition significantly influences the viscosity, handling characteristics, curing characteristics, and mechanical attributes of restorative composites [1,2]. Commonly used dimethacrylate monomers encompass bisphenol A-glycidyl methacrylate (Bis-GMA), triethylene glycol dimethacrylate (TEGDMA), and urethane dimethacrylate (UDMA), each demonstrating different rheological and polymerisation characteristics [1]. Bis-GMA-rich composites generally exhibit higher viscosity because of their rigid aromatic structure and hydrogen bonding interactions, whereas TEGDMA-rich composites contain a low-viscosity diluent monomer that enhances flowability and monomer mobility [1,2]. UDMA-rich composites demonstrate relatively flexible urethane linkages that contribute to improved polymer network formation and mechanical behaviour [2].

Preheating of resin composites has gained considerable clinical interest as an approach to improve material adaptation and handling during restorative procedures [3,4]. Raising the composite temperature lowers viscosity and increases molecular mobility, thereby enhancing the diffusion of reactive radicals during polymerisation [3,5]. Previous investigations have demonstrated that preheating may improve the degree of conversion, surface hardness, and handling properties of resin composites [6].

Degree of conversion is recognised as a critical determinant of the clinical performance and longevity of resin composite restorations [1]. Inadequate polymerisation may adversely affect wear resistance, colour stability, marginal adaptation, and biocompatibility because of residual unreacted monomers within the resin matrix [1,2]. Attenuated Total Reflectance-Fourier Transform Infrared Spectroscopy (ATR-FTIR) is a well-validated and widely accepted method for evaluating polymerisation efficiency through assessment of residual carbon-carbon double bonds [5,7,8].

Apart from polymerisation efficiency, rheological behaviour is a key determinant of the clinical handling and adaptability of restorative composites [3]. Reduction in viscosity following preheating may improve adaptation to cavity walls and decrease void incorporation during placement [3]. Mechanical properties such as microhardness are also closely related to the degree of conversion and cross-link density within the polymer network [9,10]. Increased hardness values generally indicate improved polymerisation and enhanced resistance to surface deformation [9].

Although numerous studies have reported improvements in degree of conversion, hardness, and handling characteristics following composite preheating, most investigations have focused on individual commercial materials or evaluated isolated outcome variables. Consequently, the extent to which different resin matrix chemistries influence the response to thermal preconditioning remains incompletely understood.

Resin systems based on Bis-GMA, UDMA and TEGDMA differ substantially in molecular architecture, viscosity, chain mobility and polymer network formation. These differences may significantly influence the extent to which preheating affects polymerisation efficiency, flow behaviour  and mechanical performance. However, comparative studies simultaneously evaluating degree of conversion, rheological behaviour, and microhardness among these major resin matrix systems under standardised experimental conditions remain limited [5,6].

The rationale of the present study was to comparatively evaluate the influence of preheating on resin composites with different resin matrix chemistries under standardised experimental conditions, as the response to thermal preconditioning may vary depending on monomer composition and directly influence clinical handling and mechanical performance.

Knowledge regarding the degree of conversion, rheological behaviour, and microhardness of resin composites may help clinicians select appropriate restorative materials and optimise preheating protocols to achieve improved handling characteristics, better marginal adaptation, enhanced polymerisation efficiency, and superior mechanical performance in clinical practice.

The existing literature primarily demonstrates that preheating can improve composite performance; however, it remains unclear whether these improvements occur uniformly across different resin matrix systems. Because resin chemistry directly influences viscosity, molecular mobility, and polymer network formation, comparative evaluation of different monomer systems may provide insight into material-specific responses to thermal preconditioning. This represents the primary knowledge gap addressed in the present investigation.

## Materials and Methods

This *in vitro* investigation was undertaken at the Department of Conservative Dentistry and Endodontics, AB Shetty Memorial Institute of Dental Sciences (ABSMIDS), Nitte (Deemed to be University), Deralakatte, Mangaluru, Karnataka, India. Ethics approval was secured from the Institutional Ethics Committee (Ref No: ETHICS/ABSMIDS/444/2024).

*Armamentarium*

The restorative materials evaluated in the study included Bis-GMA-rich composite (Filtek Z250, 3M ESPE), TEGDMA-rich composite (Prime Dental Restorite), and UDMA-rich composite (Tetric N-Ceram, Ivoclar). The instruments used included a Bruker Alpha II Attenuated Total Reflectance-Fourier Transform Infrared Spectrophotometer (Massachusetts, USA), dental resin composite warmer (Endoking), controlled-stress rotational rheometer, and Mitutoyo HM-200 series microhardness testing machine.

The composite materials were kept in a clean, dry setting at ambient temperature (25°C) according to the manufacturer's instructions until use.

*Grouping of Specimens*

A total of 150 specimens were prepared and divided into six experimental groups according to resin matrix composition and temperature condition (Table 1). Sample size selection was based on previously published laboratory investigations evaluating the influence of preheating on resin composite polymerisation and mechanical properties. Similar sample sizes have been reported to provide reproducible measurements and statistically meaningful comparisons for degree of conversion, rheological analysis, and microhardness testing under controlled experimental conditions.

*Preheating Procedure*

For the experimental groups, composite syringes were preheated with the Endoking composite warmer (Figure 1) at a temperature of 60°C for 10 minutes before manipulation. The temperature of 60°C was selected because previous investigations have demonstrated significant reductions in viscosity and improvements in polymerisation efficiency at this temperature while remaining within clinically acceptable thermal limits. A heating duration of 10 minutes was employed to ensure adequate thermal equilibration of the composite material before specimen preparation. The material was dispensed immediately after removal from the warming device, and all preheated materials were manipulated within a standardised time interval to minimise heat dissipation and maintain consistency among specimens.

*Specimen Preparation*

For degree of conversion analysis, composite samples were dispensed into custom-made Teflon moulds measuring 6 mm in diameter and 3 mm in thickness. The specimens were covered with Mylar strips and glass slides to ensure a uniform, smooth surface and minimise oxygen inhibition.

For rheological analysis, uncured composite material was dispensed directly onto the lower plate of a rotational rheometer operating under controlled-stress conditions with a parallel-plate geometry and a standardised 1 mm measuring gap.

For Vickers microhardness testing, cylindrical specimens measuring 6 mm in diameter and 2 mm in thickness were fabricated in stainless-steel/Teflon moulds. The specimen surfaces underwent polishing with 600-1200-grit silicon carbide abrasive paper before indentation.

*Light Curing Procedure*

All specimens were polymerised with an LED light-curing device at an irradiance of 1200 mW/cm² for a duration of 20 seconds. The curing tip maintained direct contact with the Mylar strip throughout the curing cycle. All specimens were cured by one operator to reduce procedural variability. All specimen preparation procedures and measurements were performed by a single calibrated operator to limit inter-examiner variability. Intra-examiner reliability was assessed by repeating measurements in randomly selected specimens under identical conditions.

*Degree of Conversion Analysis*

Degree of conversion was quantified using ATR-FTIR spectroscopy (Bruker Alpha II spectrophotometer) (Figure 1). The uncured and cured spectra of each composite were recorded, and the reduction in the absorbance of the aliphatic carbon–carbon double-bond peaks was analysed to calculate the degree of conversion. The degree of conversion was calculated by comparing the absorbance ratios of characteristic methacrylate peaks before and after polymerisation. Five specimens were evaluated for each experimental subgroup.

*Rheological Analysis*

Viscosity measurements were obtained under steady-shear conditions at varying shear rates to evaluate the flow behaviour of the resin composites under different temperature conditions with a controlled-stress rotational rheometer (Anton Paar, Austria) (Figure 1). The rheometer was equipped with a temperature-controlled Peltier plate and parallel-plate geometry, and a standardised measuring gap of 1 mm was maintained throughout testing. A controlled shear-rate sweep ranging from 0.1 to 100 s^-¹^ was used for viscosity measurements. Rheological parameters comprising viscosity, storage modulus (G′), and loss modulus (G″) were analysed under controlled environmental conditions. Ten independent measurements were obtained for each experimental subgroup.

*Vickers Microhardness Analysis*

Vickers microhardness analysis was performed using a Mitutoyo HM-200 series microhardness testing machine (Figure 1) under an applied load of 200 g with a dwell period of 10 seconds. Three indentations were placed at distinct sites on each specimen surface, and the average hardness value was recorded. Ten specimens were evaluated for each experimental subgroup.

*Statistical Analysis*

The sample size was determined with reference to prior *in vitro* literature examining physical and mechanical properties of resin composites under comparable conditions. The number of specimens included in each group was considered sufficient to obtain reproducible and statistically analysable data under standardised laboratory conditions. Data normality was verified using the Shapiro-Wilk test before application of parametric statistical tests. Homogeneity of variance was assessed before ANOVA.

The obtained data were analysed with SPSS software version 26.0 (IBM Corp., Armonk, NY, USA). Independent-samples Student’s t-tests were used to compare the room-temperature and preheated groups within each resin system for degree of conversion and Vickers microhardness. Intergroup comparisons were performed using one-way ANOVA with Tukey HSD post hoc testing. Two-way ANOVA examined the influence of resin type, temperature condition, and their interaction on rheological properties. A *p-value* below 0.05 was taken as the threshold for statistical significance.

## Results

*Degree of Conversion Analysis*

The degree of conversion (DC%) values obtained from ATR-FTIR analysis demonstrated improved polymerisation efficiency following preheating in all evaluated resin composite systems (Table 2).

Among the tested materials, TEGDMA-rich composites demonstrated the highest degree of conversion values under both room temperature and preheated conditions, followed by UDMA-rich composites, whereas Bis-GMA-rich composites exhibited the lowest values. Preheating increased the mean degree of conversion from 55.94 ± 0.36 to 57.68 ± 1.02 in Bis-GMA-rich composites, from 62.90 ± 0.87 to 65.87 ± 0.68 in UDMA-rich composites, and from 63.76 ± 0.85 to 66.70 ± 0.95 in TEGDMA-rich composites.

Independent-samples Student’s t-tests confirmed statistically significant increases in the degree of conversion following preheating across all resin systems. Intergroup comparison using one-way ANOVA revealed significant differences among the experimental subgroups [F(5,24)=187.4, *p<*0.001].

*Rheological Analysis*

Rheological evaluation demonstrated differences in viscosity and flow characteristics among the evaluated resin composites (Table 2). All tested materials displayed non-Newtonian shear-thinning behaviour, with viscosity progressively declining as shear rate increased.

At a shear rate of 100 s^-¹^, all evaluated composites demonstrated significant reductions in viscosity following preheating. The Bis-GMA-rich composite exhibited the highest viscosity under both temperature conditions, decreasing from 8,950 ± 540 mPa·s at room temperature to 3,420 ± 280 mPa·s after preheating. The UDMA-rich composite demonstrated intermediate viscosity values, decreasing from 4,820 ± 390 mPa·s to 1,870 ± 160 mPa·s following preheating. The TEGDMA-rich composite exhibited the lowest viscosity values under both conditions, decreasing from 1,260 ± 115 mPa·s at room temperature to 480 ± 55 mPa·s after preheating.

These findings indicate that preheating significantly improved the flowability of all evaluated resin systems. Two-way ANOVA demonstrated statistically significant effects of resin matrix composition and temperature on viscosity values (*p<*0.001).

*Vickers Microhardness Analysis*

Vickers microhardness values increased in all preheated groups when compared with the corresponding room temperature groups (Table 2). UDMA-rich composites demonstrated the highest hardness values under both room temperature and preheated conditions, whereas TEGDMA-rich composites exhibited comparatively lower hardness values.

Preheating increased the mean hardness values from 62.5 ± 3.1 to 78.7 ± 3.9 in Bis-GMA-rich composites, from 70.3 ± 3.5 to 84.7 ± 4.2 in UDMA-rich composites, and from 59.4 ± 3.0 to 72.4 ± 3.6 in TEGDMA-rich composites.

Student's t-test demonstrated statistically significant increases in Vickers microhardness following preheating in all evaluated resin systems (*p<*0.001). Intergroup comparison using one-way ANOVA confirmed statistically significant differences across all experimental groups [F(5,54)=68.3, *p<*0.001].

Figure 2 demonstrates the comparison of degree of conversion and Vickers microhardness values among the evaluated resin composite groups under room temperature and preheated conditions. Increases in both the degree of conversion and microhardness were observed following preheating in all resin systems. UDMA-rich composites demonstrated the highest microhardness values, whereas TEGDMA-rich composites demonstrated the highest degree of conversion values. 

## Discussion

The present *in vitro* study assessed the influence of preheating on the degree of conversion, rheological behaviour, and Vickers microhardness of resin composites with different resin matrix compositions. The findings demonstrated that preheating significantly influenced the polymerisation efficiency, flow characteristics, and mechanical attributes of the evaluated composites.

Preheating produced an elevation in the degree of conversion values in all tested resin systems. Increased composite temperature enhances molecular mobility and reduces viscosity, thereby facilitating improved diffusion of free radicals during polymerisation [3,5]. The current results are consistent with prior investigations that demonstrated improved degree of conversion following preheating of resin composites [6,7]. Preheating promotes greater monomer conversion by increasing chain mobility and reducing steric hindrance during the polymerisation reaction [5].

Among the evaluated materials, TEGDMA-rich composites demonstrated the highest degree of conversion values under both room temperature and preheated conditions. TEGDMA is a relatively low-viscosity monomer with greater molecular flexibility, allowing improved monomer mobility and enhanced polymerisation efficiency [1,2]. In contrast, Bis-GMA-rich composites demonstrated comparatively lower degree of conversion values, which may be attributed to the rigid aromatic backbone and increased intermolecular hydrogen bonding associated with Bis-GMA monomers [1,2]. These characteristics increase viscosity and may limit monomer movement during polymerisation [1].

The rheological analysis demonstrated differences in viscosity and flow properties among the evaluated resin composites. All tested materials showed non-Newtonian shear-thinning behaviour, consistent with the viscoelastic nature of resin-based restorative materials [3]. Preheating influenced the viscosity and flow behaviour of the evaluated resin composites, indicating temperature-dependent changes in rheological properties. Reduction in viscosity following preheating has been previously reported and is attributed to heightened molecular motion and weakened intermolecular interactions within the resin matrix [3,5].

The marked reduction in viscosity observed after preheating may explain the corresponding improvements in degree of conversion. Increased molecular mobility facilitates diffusion of reactive species and promotes more efficient polymer network formation. Consequently, rheological changes induced by thermal conditioning appear closely linked to the polymerisation behaviour observed in the present study.

In the present study, all evaluated resin composites demonstrated a significant reduction in viscosity following preheating. Bis-GMA-rich composites exhibited the highest viscosity values under both temperature conditions, whereas TEGDMA-rich composites demonstrated the lowest viscosity values. These findings are consistent with the inherent molecular characteristics of the respective monomer systems. The rigid aromatic backbone and extensive hydrogen bonding associated with Bis-GMA contribute to higher viscosity, whereas the lower molecular weight and greater flexibility of TEGDMA promote improved flowability. UDMA-rich composites demonstrated intermediate viscosity values, reflecting their comparatively flexible urethane-based molecular structure. Importantly, the present findings suggest that the influence of preheating is dependent on resin matrix chemistry. Although all tested materials demonstrated improved polymerisation followingpre heating, the magnitude of improvement differed among resin systems. This observation indicates that monomer composition plays a significant role in determining the effectiveness of preheating and supports the concept of a material-specific response rather than a universal temperature effect [1,2].

Improved flow behaviour following preheating may enhance adaptation of restorative composites to cavity walls and reduce the incorporation of voids during placement [3]. Enhanced adaptation may contribute to improved marginal integrity and reduced microleakage in clinical situations. Similar findings regarding improved adaptation and reduced gap formation following preheating have been reported previously [11,12]. However, excessive temperature elevation may also increase the rate of polymerisation shrinkage and intrapulpal temperature rise [4,10]. Previous studies have demonstrated that excessive thermal rise during restorative procedures may adversely affect pulpal tissues [13-15]. Zach and Cohen reported that temperature increases beyond critical thresholds may result in irreversible pulpal injury [15]. Nevertheless, the preheating temperature used in the present study was within clinically accepted limits reported in previous investigations [4,15].

Vickers microhardness values increased significantly following preheating in all evaluated resin systems. Microhardness is considered an indirect indicator of polymerisation efficiency and cross-link density within the polymer matrix [9,10]. Increased hardness recorded after preheating may therefore be explained by greater monomer conversion and a denser polymer network [5,9]. The present findings are comparable with the results of Elkaffas *et al*. [9], Singha *et al*. [10], and Dionysopoulos *et al*. [16], who observed improved microhardness and polymerisation behaviour following preheating of resin composites. Similarly, Lohbauer *et al*. [5] reported enhanced monomer conversion after thermal preconditioning of restorative composites.

Among the tested materials, UDMA-rich composites demonstrated the highest microhardness values under both temperature conditions. UDMA monomers exhibit relatively flexible molecular structures that facilitate efficient cross-linking and improved mechanical behaviour [2]. In contrast, TEGDMA-rich composites demonstrated comparatively lower hardness values despite exhibiting higher degree of conversion. This observation may be related to the lower molecular weight and greater flexibility of TEGDMA-rich polymer networks, which may reduce resistance to surface indentation [1,2].

The outcomes of this study align with earlier investigations and systematic reviews reporting improvements in physical and mechanical properties when resin composites are preheated [6,9,17]. The results further support the clinical use of preheating as a method to improve handling characteristics and polymerisation behaviour of restorative composites.

From a clinical perspective, the observed reduction in viscosity may improve adaptation of restorative materials to cavity walls and decrease void incorporation during placement. Likewise, the increases in the degree of conversion and microhardness may contribute to enhanced wear resistance, improved surface durability and greater resistance to degradation. Although the absolute improvements observed in the present study were moderate, their cumulative effect may be clinically beneficial, particularly in restorations subjected to long-term functional loading.

Certain limitations should be considered while interpreting the findings of the present study. This investigation was performed under controlled laboratory conditions that may not fully replicate the intraoral environment. Parameters such as thermal cycling, masticatory loading, salivary exposure, and extended ageing were outside the scope of this study. In addition, only selected resin matrix systems and a single preheating temperature were investigated. Further long-term clinical and laboratory studies are recommended to examine the impact of varying preheating temperatures and ageing conditions on restorative composite performance. Subsequent research should assess long-term ageing behaviour, thermal cycling, wear resistance, and clinical outcomes of preheated resin composites under simulated intraoral conditions.

Within the limitations of this *in vitro* investigation, preheating at 60°C improved the degree of conversion, rheological behaviour, and microhardness of all evaluated resin composites. However, the magnitude of improvement varied according to resin matrix composition, indicating that the response to thermal conditioning is material-dependent. These findings contribute to the understanding of how resin chemistry influences the effectiveness of composite preheating and may assist clinicians in optimising restorative material selection and handling protocols.

## Conclusions

Within the scope and constraints of the present *in vitro* investigation, preheating of resin composites at 60°C enhanced the degree of conversion, rheological behaviour, and Vickers microhardness of the evaluated materials. TEGDMA-rich composites demonstrated the highest degree of conversion, whereas UDMA-rich composites exhibited superior microhardness values. Preheating may therefore be considered a beneficial clinical approach for improving the handling characteristics and mechanical performance of restorative resin composites.

## Figures and Tables

**Table 1:** Table 1: Distribution of experimental groups and specimens according to resin matrix composition, temperature condition, and experimental tests.

**Group**	**Resin System**	**Temperature Condition**	**Degree of Conversion (n)**	**Rheological Analysis (n)**	**Vickers Microhardness (n)**
Group I	Bis-GMA-rich composite	Room temperature (25°C)	5	10	10
Group II	Bis-GMA-rich composite	Preheated (60°C)	5	10	10
Group III	UDMA-rich composite	Room temperature (25°C)	5	10	10
Group IV	UDMA-rich composite	Preheated (60°C)	5	10	10
Group V	TEGDMA-rich composite	Room temperature (25°C)	5	10	10
Group VI	TEGDMA-rich composite	Preheated (60°C)	5	10	10

**Table 2:** Table 2: Degree of conversion, rheological analysis, and Vickers microhardness values of resin composites under room temperature and preheated conditions.

**Resin System**	**Degree of Conversion (%) RT**	**Degree of Conversion (%) Preheated**	**Viscosity RT (mPa·s)**	**Viscosity Preheated (mPa·s)**	**Vickers Microhardness RT (HV)**	**Vickers Microhardness Preheated (HV)**
Bis-GMA-rich composite	55.94 ± 0.36	57.68 ± 1.02	8950 ± 540	3420 ± 280	62.5 ± 3.1	78.7 ± 3.9
UDMA-rich composite	62.90 ± 0.87	65.87 ± 0.68	4820 ± 390	1870 ± 160	70.3 ± 3.5	84.7 ± 4.2
TEGDMA-rich composite	63.76 ± 0.85	66.70 ± 0.95	1260 ± 115	480 ± 55	59.4 ± 3.0	72.4 ± 3.6

Values are expressed as mean±standard deviation (SD). RT, room temperature. Independent-samples Student’s t-tests, one-way ANOVA and two-way ANOVA were used as appropriate. Statistical significance was set at p<0.05.

**Figure 1:**
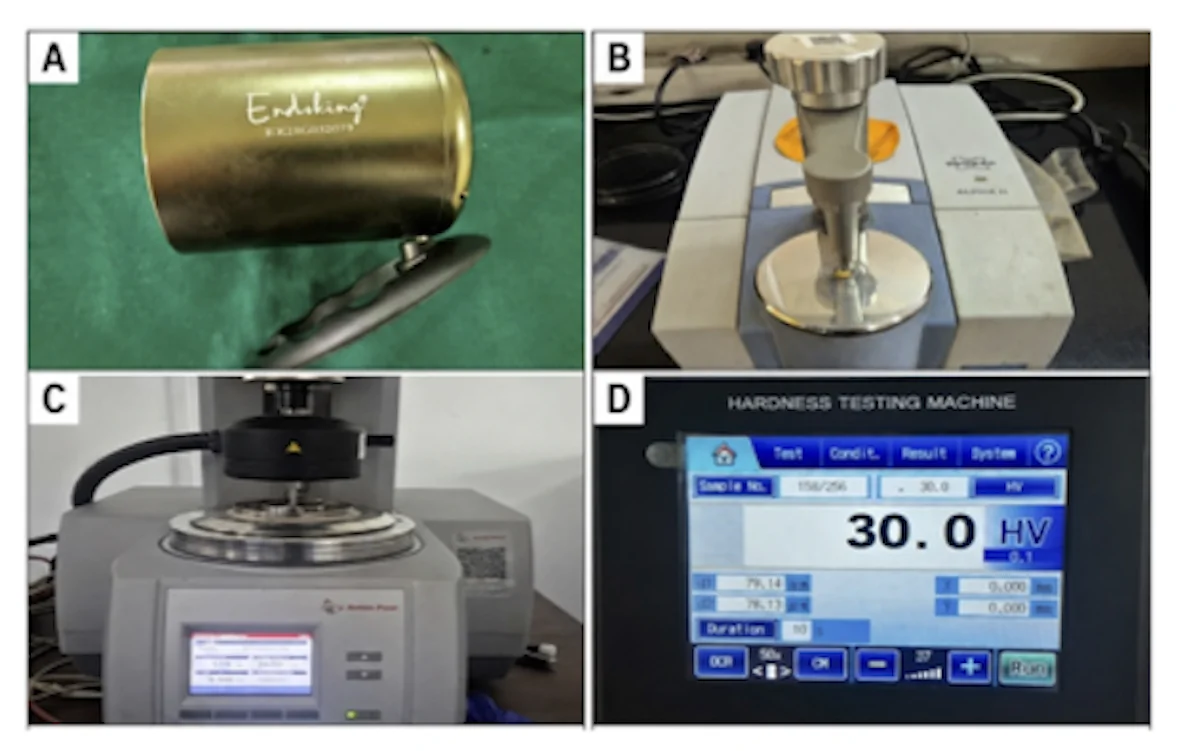
Figure 1: Experimental setup used in the study. A: Composite warmer (Endoking). B: Attenuated total reflectance-Fourier transform infrared spectroscopy (ATR-FTIR). C: Rotational rheometer. D: Vickers microhardness testing machine.

**Figure 2:**
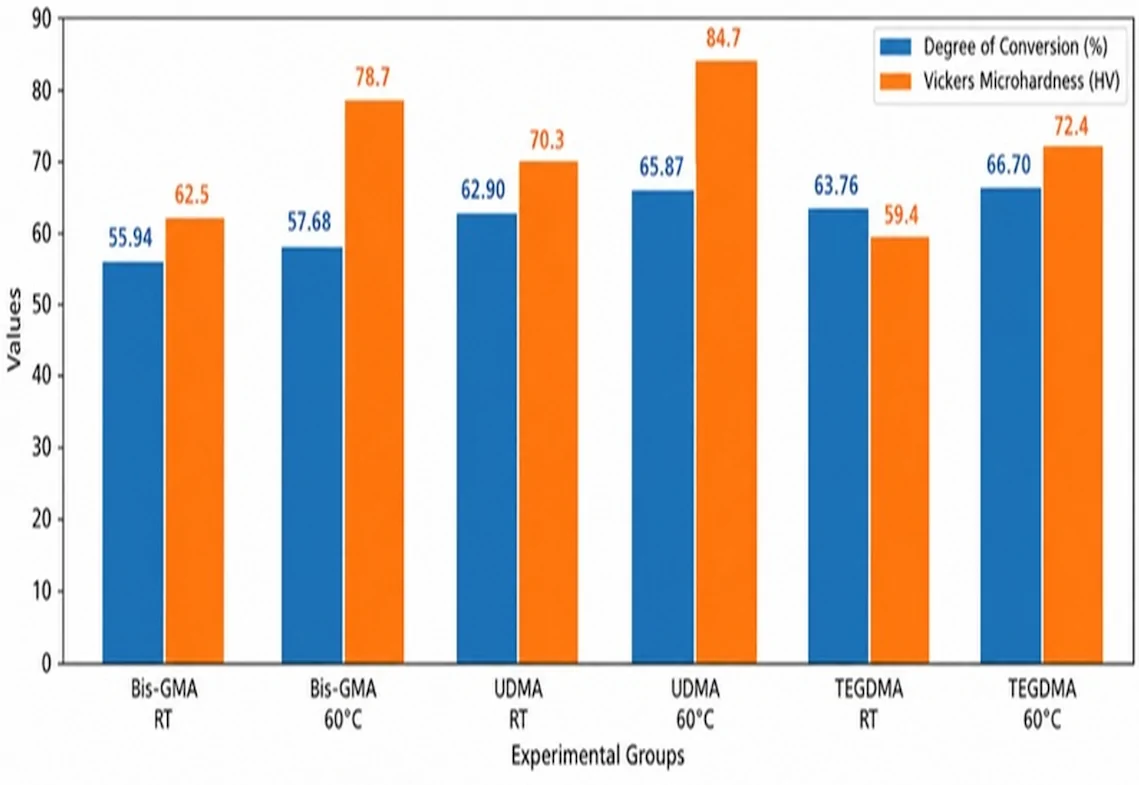
Figure 2: Comparison of degree of conversion and Vickers microhardness values between room temperature and preheated resin composite groups.

## Data Availability

none.
